# Impact of an Internet-Delivered Sound Healing Intervention on Chronic Non-Malignant Pain and Sleep Disturbances in Community Settings

**DOI:** 10.1192/j.eurpsy.2024.367

**Published:** 2024-08-27

**Authors:** N. Sharma, D. Walker, B. S. Prasad, M. Patel

**Affiliations:** ^1^Aarogyam UK CIC, Leicester; ^2^Suara Sound Academy, Callington, United Kingdom

## Abstract

**Introduction:**

Chronic pain patients often contend with insomnia symptoms, creating a reciprocal relationship that adds complexity to their condition. Evaluating interventions targeting insomnia in this population becomes paramount, given the intertwined nature of pain and sleep disturbances.

**Objectives:**

This retrospective pretest design aimed to assess the efficacy of an Internet-delivered sound healing intervention in reducing insomnia severity and addressing sleep- and pain-related parameters among individuals with chronic pain.

**Methods:**

Conducted as a community-based project, Tuning for Health provided support to individuals grappling with long-term illnesses. The intervention involved the virtual delivery of a specially crafted sound track using tuning forks over a 6-week period, supervised by an experienced therapist and administered weekly for an hour. Participants were instructed to play the track daily at a time convenient for them. A total of 68 participants (mean age 59.3 years) completed the intervention. Outcome measures, including the Insomnia Severity Index (ISI), a sleep diary, and assessments for anxiety, depression, and pain-related parameters, were collected at the end of the 6-week intervention and repeated after a 6-month follow-up. Negative effects were monitored and reported.

**Results:**

Significant immediate interaction effects (time by treatment) were observed for the pain severity, ISI and various sleep parameters, such as sleep efficiency, sleep onset latency, early morning awakenings, and wake time after sleep onset. A time effect for anxiety and depression was noted at the 6-month follow-up. The group exhibited highly significant improvements in pain-related parameters. At the 6-month follow-up, sustained enhancements in sleep parameters and mental health were reported, with no reported side effects.

**Conclusions:**

These unique results suggest the potential efficacy of sound healing in alleviating chronic pain and associated insomnia. Further research with a larger sample size is warranted to validate these findings. Combining sound healing with other treatments may offer enhanced outcomes for individuals dealing with both chronic pain and comorbid insomnia. This study lays the groundwork for future investigations into the promising intersection of sound healing, chronic pain management, and sleep improvement.

**Disclosure of Interest:**

None Declared

